# Male Sex Hormones, Metabolic Syndrome, and Aquaporins: A Triad of Players in Male (in)Fertility

**DOI:** 10.3390/ijms24031960

**Published:** 2023-01-19

**Authors:** Diana C. Nunes, João C. Ribeiro, Marco G. Alves, Pedro F. Oliveira, Raquel L. Bernardino

**Affiliations:** 1 UMIB—Unit for Multidisciplinary Research in Biomedicine, ICBAS—School of Medicine and Biomedical Sciences, University of Porto, Rua Jorge Viterbo Ferreira 228, 4050-313 Porto, Portugal; 2 ITR—Laboratory of Integrative and Translocation Research in Population Health, Rua das Taipas 135, 4050-600 Porto, Portugal; 3 LAQV-REQUIMTE, Department of Chemistry, University of Aveiro, 3810-193 Aveiro, Portugal

**Keywords:** male infertility, testosterone, hypogonadism, metabolism, hormonal imbalance, osmoregulation

## Abstract

Infertility is becoming a chronic and emerging problem in the world. There is a resistant stigma that this health condition is mostly due to the female, although the literature supports that the responsibility for the onset of infertility is equally shared between both sexes in more or less equal proportions. Nevertheless, male sex hormones, particularly testosterone (T), are key players in male-related infertility. Indeed, hypogonadism, which is also characterized by changes in T levels, is one of the most common causes of male infertility and its incidence has been interconnected to the increased prevalence of metabolic diseases. Recent data also highlight the role of aquaporin (AQP)-mediated water and solute diffusion and the metabolic homeostasis in testicular cells suggesting a strong correlation between AQPs function, metabolism of testicular cells, and infertility. Indeed, recent studies showed that both metabolic and sexual hormone concentrations can change the expression pattern and function of AQPs. Herein, we review up-to-date information on the involvement of AQP-mediated function and permeability in men with metabolic syndrome and testosterone deficit, highlighting the putative mechanisms that show an interaction between sex hormones, AQPs, and metabolic syndrome that may contribute to male infertility.

## 1. Introduction

Infertility is defined as the inability to obtain a clinical pregnancy after 12 months or more of frequent unprotected intercourse, according to the World Health Organization (WHO) criteria [1]. Estimations show that around 48 million couples and 186 million reproductive-aged individuals are infertile worldwide, however more recent statistics are worrisomely lacking [2]. The male factor is reported to be responsible for 50% of all infertility cases and is present in 20–30% of infertile couples [3]. Additionally, testicular activity abnormalities and/or hormonal imbalances account for two out of three cases of male infertility [3,4].

Testosterone (T), the so-called male gonadal hormone, is vital in terms of sexual and reproductive function, as well as bone health, body composition, and behavior [5]. All of this led to the assumption that this hormone acts at levels that go beyond the male reproductive system, being able to interact with most tissues. In this regard, one of the most discussed and highlighted interactions in the literature is the pathological relation between T deficit, lifestyle, and metabolic disorders [6]. Several studies have reported a clear association between metabolic diseases and hypogonadism, namely with the hypogonadotropic (dysregulation on the secretion of hormones that stimulate the testis) and late-onset subtypes (develops at a later age) [7,8,9]. Dysregulation of hormone secretion is known to modulate the permeability of the pores responsible for the permeability of water and solutes like glycerol, a crucial part of cellular function [10,11]. Aquaporins (AQPs) are the membrane channels responsible for the transport of water and other solutes. Aquaglyceroporins (AQGPs), a subfamily of these transmembrane pores, are thought to have an important role in lipid metabolism, modulating both lipolysis and lipogenesis [10,11]. Thus, abnormal AQP-mediated permeability is suggested to be intrinsically involved in the development of metabolic diseases (for review see [12]). Likewise, proper AQP-mediated water and glycerol diffusion is vital for spermatogenesis, spermiogenesis, and sperm maturation (for review see [13]). The objective of this review is to highlight the connection between both sexual and metabolic hormone secretion patterns and their effect on AQP-mediated water and solute diffusion on the onset of male (in)fertility.

## 2. Testosterone and Spermatogenesis

Despite the diverse functions in both males and females [14], T is known as the primary male sex hormone. T belongs to a particular class of steroid hormones called androgens. These are synthesized in the adrenal glands and gonads and are responsible for the development of sexual characteristics and play a key role in the maintenance of reproductive health [15,16,17]. Additionally, T and other androgens can undergo a process of aromatization, giving rise to 17-β estradiol (E2), another crucial hormone in the male reproductive context, namely in terms of modulating libido, erectile function, and spermatogenesis [18].

**Figure 1:**
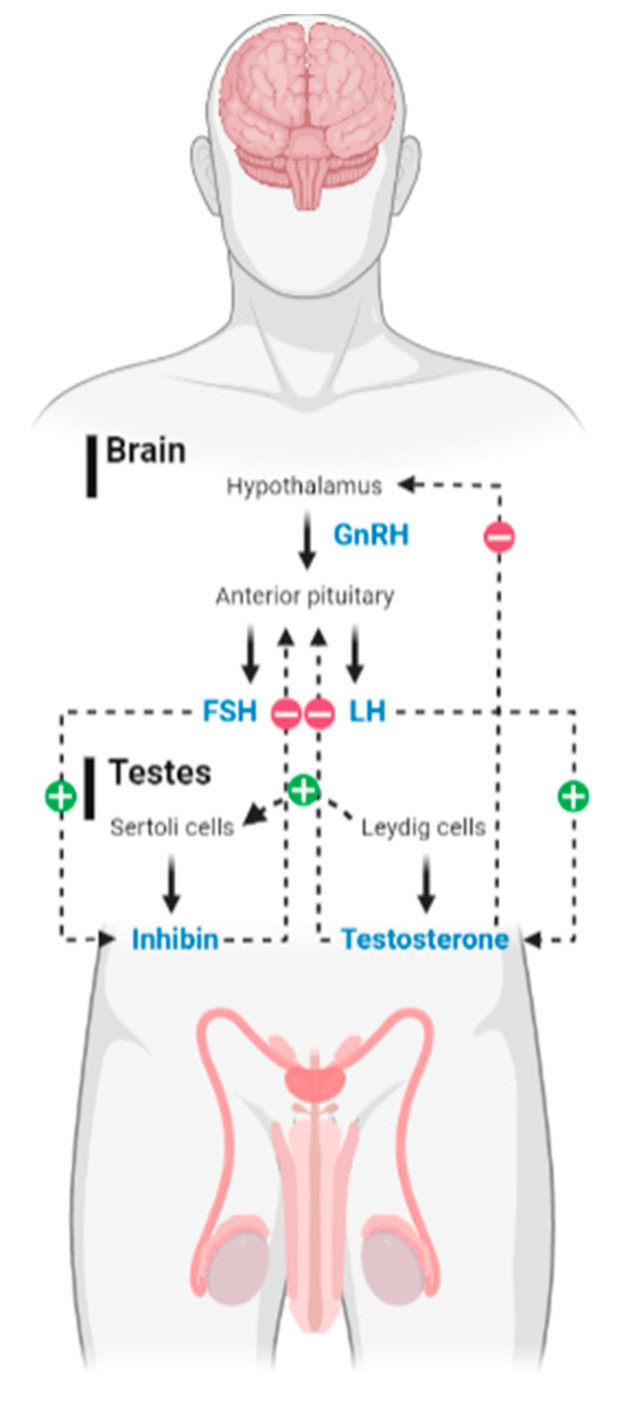
Overview of the hormonal interaction in hypothalamus-pituitary-gonadal (HPG) axis. The hypothalamic-pituitary-gonadal (HPG) axis boils down to a highly regulated system whose main function is to ensure androgen biosynthesis and promote spermatogenesis. This process revolves around a hormonal balance. As such, it simultaneously requires the generation of stimulatory and inhibitory impulses. In this image, the bold arrows indicate the secretion of hormones; the dotted arrows represent the stimulating (+) or inhibitory (−) effect of the secreted hormones. In short, the hypothalamus generates pulses of gonadotropin-releasing hormone (GnRH) that reach the pituitary, inciting it to produce follicle-stimulating hormone (FSH) and luteinizing hormone (LH). In turn, these glycoproteins act on the Sertoli cells (SCs) and the Leydig cells (LCs), respectively. This interaction results in the production of other hormones, such as testosterone by LCs and inhibin by the SCs, which generate negative feedback impulses to the central command of the axis, thus maintaining a balance of hormone synthesis and secretion. LCs also have some mechanisms that allow them to stimulate SCs directly.

Spermatogenesis can be defined as a process of cell differentiation that leads to the production of mature and fertilizable sperm. It is a highly complex process, controlled by several hormonal cascades that influence male reproductive potential. The hypothalamic-pituitary (HP) axis is considered to be responsible for the initiation of many hormonal changes necessary for many bodily functions in distinct organs [19]; however, we will focus on the one most responsible for male fertility [20]—the hypothalamic–pituitary–gonadal (HPG) axis. This system is known to be regulated through negative feedback mechanisms [21] (Figure 1). In brief, internal or external stimuli reach the central system and act on the hypothalamus that, subsequently, initiates a process of integration of these signals. This results in the expression of kisspeptins, which are responsible for the induction of gonadotropin-releasing hormone (GnRH) secretion by the hypothalamus [22,23]. When stimulated, the anterior pituitary produces and secretes FSH and LH, which enter the systemic circulation to act on the endocrine cells located in the gonads, inciting the production of T and E2. LH acts as the main differentiator and proliferator of Leydig cells (LCs) [24] (which occupy 10–20% of the interstitial compartment of the testis [25]), being also the main actor responsible for inciting the production of T in these cells [26]. In turn, T diffuses into the seminiferous tubules, where spermatogenesis and all related processes occur. On the other hand, it is known that FSH is able to trigger spermatogenesis at puberty [27], being also responsible for the maintenance and regulation of this process throughout adulthood, as well as for the preservation of testicular size and the number of viable spermatozoa [17,28]. Both FSH and T bind to specific receptors expressed on Sertoli cells (SCs) [24,27], which are somatic testicular cells responsible for ensuring a proper ionic and nutritional environment for spermatogenesis, and also for ensuring the integrity of the blood-testis barrier [24].

T enters the circulation mostly in its inactive form, i.e., associated with serum albumin or sex hormone-binding globulin [29], and its action is indirectly mediated through its conversion to dihydrotestosterone (DHT) by 5-α-reductase or to E2 by aromatase, or directly, through its binding to androgen receptors (AR). Consequently, E2 binds to estrogen receptors, inhibiting the kisspeptin-producing neurons, kisspeptin/neurokinin B/dynorphin, thus ceasing the production of FSH and LH by blocking the action of GnRH [30], closing the cycle.

T acts in both classical and non-classical routes in SCs [31,32]: by direct changes in gene expression; or via activation of kinases that may be involved in vital processes related to spermatogenesis [33]. Throughout the classical T signaling route, T diffuses across the plasma membrane and interacts with the AR. In turn, AR will reach the nucleus and interact with certain DNA sequences known as androgen response elements, modulating gene expression, and impacting cellular function [31,32,33]. In addition to the classic route, there are at least two significant non-classical T action pathways in SCs. T interacts with the AR in the non-classical kinase activation route, which enables it to draw in and activate the protein tyrosine kinases, which in turn activates the epidermal growth factor receptor through an intracellular pathway involving the mitogen-activated protein kinase (MAPK) cascade and epidermal growth factor receptor, most likely through Ras proteins [34]. Another non-classical route involves T interacting with a plasma membrane receptor that resembles a Gq coupled G-protein coupled receptor and the consequent lowering of phosphatidylinositol 4,5-bisphosphate (PIP2) concentration enables cytosolic Ca^2+^ increase through K+-ATP channels blockage [31,32,34].

## 3. Consequences of Sexual Hormone Dysregulation to Male Fertility

Either through classical or non-classical pathways, T takes a crucial role in spermatogenesis, a process that proves to be, by itself, a highly complex event, controlled by several hormonal cascades that anatomically and physiologically influence male reproductive potential. In response to the regulation of GnRH secretion by the hypothalamus, testicular activity is mainly inhibited by a negative feedback signal led by the inhibin produced by SCs, and by E2 [26]. When this response is affected, the maintenance of male reproductive and sexual function is compromised, and this impairment can arise at several levels. For example, the literature suggests that high concentrations of E2 promote the blockade of the HPG axis [18,35]. On the other hand, abnormal secretion of metabolic hormones such as leptin also dysregulates GnRH secretion, resulting in inhibited steroidogenesis [36,37]. This fact helps to highlight the link between endocrine dysregulation and hypogonadism and strengthens the relationship between hypogonadism and other comorbidities such as metabolic diseases.

### 3.1. Male Hypogonadism

Male hypogonadism is one of the most common endocrinologic disorders. Usually, the diagnosis is made based on the symptomatology and serum morning T levels of the man [38]. The symptoms associated with male hypogonadism vary according to the severity of the hormone deficit and the age of the individual, as well as with the level of sensitivity to T [24], ranging from erectile dysfunction (ED), low sexual performance, decreased libido and frequency of morning erections, to loss of body hair, fatigue, irritability, depression, sleep disorders, and difficulty concentrating [39]. Additionally, the causes of this hormonal deficit may have diverse origins within the HPG axis. Thus, several sub-classifications of the disease arise.

#### 3.1.1. Primary Hypogonadism versus Secondary Hypogonadism

**Figure 2:**
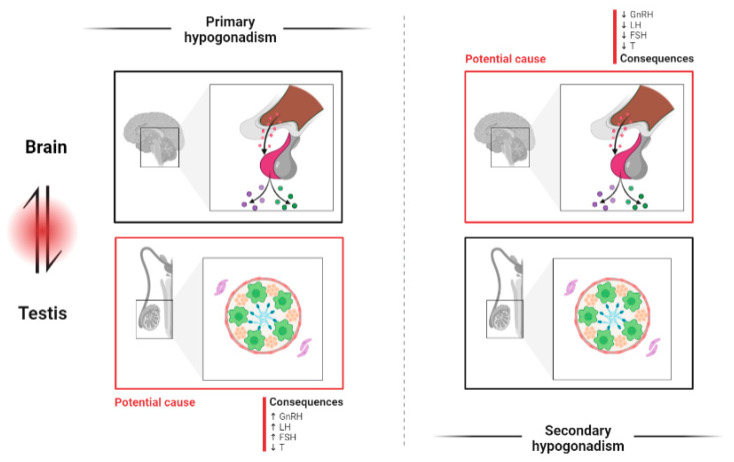
Pathological differentiation of the main types of hypogonadism in males. Male hypogonadism can be triggered either by low or by no testosterone. This phenomenon can, in turn, be triggered either in a direct and indirect way. Mentioning a direct problem in testosterone production is the same as referring to primary hypogonadism (or hypergonadotropic hypogonadism), characterized by testicular defects. On the other hand, indirect problems in testosterone production mostly concern secondary hypogonadism (or hypogonadotropic hypogonadism), characterized by imbalances in the functioning of the hypothalamus-pituitary-gonadal (HPG) axis. This image illustrates the main differences between both types of hypogonadism. The double arrow between “brain” and “testis” represents the interaction in HPG axis. The red spot is meant to illustrate the imbalance that underlies the onset of hypogonadism. The red box represents the etiological focus of the pathology according to the main types of hypogonadism, i.e., primary and secondary. In the top box, the central branch of the HPG axis is illustrated: the brain. In it, the brown area, which represents the hypothalamus, and the pink area, which represents the pituitary, can be seen. The black arrow followed by the pink balls illustrates the release of gonadotropin-releasing hormone (GnRH). The black arrow followed by the purple balls illustrates the release of luteinizing hormone (LH). The black arrow followed by the green balls illustrates the release of follicle-stimulating hormone (FSH). In the bottom box are illustrated the male gonads, i.e., the testes. In turn, the testes consist of a tight tangle of seminiferous tubules. Within these tubules, several components can be distinguished, which extend from the tubular lumen to the interstitial medium. In the lumen are the Leydig cells (LCs), represented in purple, whose function depends mostly on LH signaling. On the other hand, inside the seminiferous tubules (delimited by a layer of connective tissue, represented by the cells in red) there are the germ line cells (represented in orange), the germ cells themselves (represented in blue), and the SCs (represented in green). Similar to the LCs, the functioning of the SCs is also hormone-dependent, mainly in the signaling of FSH.

There are two main types of male hypogonadism: primary and secondary. Primary hypogonadism is characterized by a testicular failure to produce T, which arises due to an inability to respond to the gonadotropins, FSH and LH [40,41]. As such, individuals with this type of hypogonadism have high levels of FSH and LH but, in contrast, low levels of T [40] (Figure 2). On the other hand, secondary hypogonadism arises due to an endocrine dysfunction of the HP axis [42] which, in turn, generates failures in the production of GnRH. Meanwhile, that event will compromise the secretion of FSH and LH, translating into low levels of these two hormones and, consequently, low levels of T [41]. It is also important to point out that, contrary to what its etiology seems to suggest, this type of hypogonadism does not arise only as a consequence of the appearance of comorbidities associated with the inevitable senescence phenomenon, being detected in individuals of the most varied age groups [43].

#### 3.1.2. Other Classifications for Male Hypogonadism 

Adult-onset hypogonadism or late-onset hypogonadism is another class of male hypogonadism associated with its appearance at a later age [7,8,9]. The pathological aspect of the disease appears due to the manifestation of other pathologies, namely chronic and metabolic diseases, which begin to manifest at the onset of adulthood. Because it is related both to a direct decrease in T secretion and impaired steroidogenesis [44], and to a decrease in GnRH pulses [45], late-onset hypogonadism is seen as a type of combined hypogonadism, which simultaneously combines characteristics of primary and secondary hypogonadism. Furthermore, according to the literature, there are reports of the appearance of a rare form of hypogonadism, which phenotypically manifests itself through total or partial insensitivity to androgens [38].

In addition to the different types of hypogonadism already mentioned, there are other forms of etiological classification of this pathology. For example, Bhasin et al. suggest that this disease should be divided according to the organic or functional origin of the problem that triggers it [46], where organic etiology is understood as local, static, and irreversible damage (such as physical damage at the HPG level generated by trauma, cancer and/or radiation) and functional etiology is understood as one or more systemic and reversible damage (usually associated with other diseases linked to factors such as environmental contaminants and lifestyle) [42,46].

## 4. Influence of Testosterone Dysregulation on Metabolic Changes

Indeed, current data indicates that hypogonadism is influenced by environmental and lifestyle factors that trigger the onset of metabolic and reproductive dysregulations. According to the literature, metabolic diseases are also negatively associated with infertility [47,48,49]. Since hypogonadism is closely related to endocrine disorders [50,51,52,53,54], it would be expected that both metabolic diseases (such as obesity and insulin resistance, responsible for triggering the onset of T2D, gestational diabetes, and pre-diabetes) and some non-pathological conditions related to them (such as overweight) would have a pathophysiological repercussion on hypogonadism. In fact, a bidirectional link between metabolic disorders and hypogonadism has been suggested since on one hand metabolic complications have the capacity to induce hypogonadism [20,55] and on the other hand, hypogonadism can worsen the state of certain metabolic diseases [56,57,58,59,60,61,62,63,64,65]. However, this is only the tip of the iceberg when it comes to the impact of T on body composition. Numerous studies show that, regardless of age group [66], there is an inverse relationship between T levels and obesity [67,68,69,70,71]. This is evident not only in the decreased T levels in the context of the disease but also in the increase in T levels when therapies are applied, ranging from a simple diet to physical exercise, culminating in more invasive methods such as bariatric surgery [72]. Additionally, the literature indicates that middle-aged individuals suffering from type 2 diabetes (T2D) show an inverse relationship between T levels and carotid intimal thickness [73], with individuals possessing low T being more likely to develop atherosclerotic plaques and cardiovascular problems. 

In fact, it is easy to pinpoint the interaction between T and obesity discussed from a molecular perspective. For example, adipocytes express high levels of aromatase, the enzyme responsible for the conversion of T into E2. Thus, increased adipose tissue, as seen in obesity patients, translates into enhanced T aromatization and a consequent decrease in T circulation. Tumor necrosis factor α (TNF-α) and interleukin 6 (IL6) also play a role as adipocytokines, having the ability to suppress GnRH secretion [74,75,76]. On the other hand, it is known that leptin, which plays a key role in processes such as appetite regulation and maintenance of energy demand and consequent body weight control, can stimulate the brain in a manner contrary to TNF-α and IL6, inciting it to secrete GnRH. This will, in turn, stimulate the release of LH through the action of the neuroproteins kisspeptins, leading to the subsequent production of T by the testis [77]. In this sense, since increased adiposity promotes an increase in circulating leptin, one would expect T release to occur at an abnormally increased rate. However, when faced with these changes, the body adopts a negative feedback mechanism to maintain homeodynamics. Thus, leptin becomes inhibitory to T production due to the resistance of neuronal receptors to its hormonal action, but also due to its ability to directly suppress T production through its action on LCs [78]. Adiponectin is another hormone whose secretion has been closely related to that of T. Some reports show that T replacement therapy (TRT), one of the main therapeutic approaches adopted in the context of T deficit, can reduce adiponectin levels after 6 months [79,80,81]. One of the most pointed factors to justify this phenomenon is the reduction in fat mass implied in the decrease in waist circumference that occurs due to the decline in circulating adiponectin [80]. More recently, results have been published from a study that compared adiponectin levels in diabetics with and without ED [82]. These results showed that, in addition to both adiponectin and T being below normal parameters in diabetic subjects with ED when compared to diabetic subjects without ED, there is also a direct correlation between these two hormones. Hence, the complexity of this hormonal network narrated above highlights the importance of unraveling the physiological mechanisms that underlie it. Among those mechanisms, AQP-mediated water and solute diffusion appears to play a key role. Evidence suggesting this hypothesis will be presented in the next topic.

## 5. Aquaporin-Mediated Osmoregulation Role in Metabolic Diseases

Metabolic diseases are involved in an extensive network of causes and effects and, in addition to hormonal changes, there are many other changes related to these diseases, such as those related to membrane transport events. Some of the membrane transporters that may have their function altered are AQPs. So far, 13 different AQPs (0–12) have been described in mammals, found throughout virtually the entire organism, from muscle to the pancreas, adipose tissue, brain, lung, immunological system, eye, kidney, skin, gastrointestinal tract, uterus, ear, and testis [83]. Metabolically speaking, the role of AQPs becomes even more evident when we restrict the discussion to the AQGPs (Table 1), the subfamily of AQPs that, as the name implies, is mainly dedicated to the membrane transport of water and glycerol [84,85,86]. Glycerol is the functional core of the glycerol-3-phosphate shuttle, a biochemical system that crosses lipid and glucose metabolism with oxidative phosphorylation. AQGPs have a crucial role in the diffusion of glycerol through the membrane of different cell types, regulating this molecule dynamics in lipolysis and triglyceride synthesis. AQGPs also regulate the glycerol exchange between adipose tissue and the liver [87]. AQP3, AQP7, and AQP9 are expressed in adipocytes and assist in glycerol accumulation during triglyceride formation or in glycerol efflux after triglyceride catabolism [10]. On the other hand, AQP9 is expressed in hepatocytes and assists in glycerol uptake for subsequent accumulation in the form of triglycerides [11]. Thus, AQGPs are central to maintaining a healthy equilibrium of glycerol uptake or efflux (and hence for lipid metabolism). Under conditions of metabolic impairment, AQGPs expression on cellular membranes can be compromised and disrupt this equilibrium. As an example, AQP7 deficiency in adipose tissue was found to precede lipid accumulation and adipose tissue enlargement due to reduce glycerol efflux, setting the stage for the development of obesity in mice [88,89]. Another indication for the impact of metabolic diseases on AQGP expression, or vice-versa, can be highlighted by the effect that metabolic hormones have on the expression patterns of such proteins. Insulin was found to upregulate the expression of all the mentioned AQGPs (AQP3, AQP7, and AQP9) in human adipocytes and hepatocytes [90]. Notably, leptin was found to be more selective, by upregulating AQP3 expression but down-regulating AQP7 and AQP9 expression in both adipocytes and hepatocytes [90]. This study by Rodríguez and collaborators showed for the first time, that the membrane expression of AQGPs is regulated by the phosphoinositol-3-kinase (PI3K)/protein kinase B (AKT)/mammalian target of rapamycin (mTOR) signaling cascade [90]. Concurrently, it was found that the expression of AQGPs also influences metabolic hormone sensitivity. AQP7 overexpression was found to increase insulin sensitivity in insulin-resistant mice adipocytes thus, increased lipolysis by enhancing glycerol secretion [91]. 

Despite being apparent that metabolic diseases are accompanied by abnormal AQGPs expression, there is new evidence showing that metabolic diseases can be worsened by AQGPs dysfunction. Similarly, hormones involved in the HPG axis can also worsen metabolic diseases and some evidence show that the expression of AQGPs could also be involved in such events. As referred, AQGPs were found to be important for glycerol and water permeability in a plethora of cell types [10,11,35,103,104] including those of male reproductive tissues [35,96,105]. The healthy testis can maintain a controlled glycerol concentration allowing the proper development of spermatogenesis. When glycerol dynamics are altered and its concentration increases, it can disrupt the blood-testis barrier and impair the spermatogenic process [106]. Glycerol plasma concentration is known to be higher in obese, obese diabetic, and non-obese diabetic men [107,108]. Ideally, this increase in circulating glycerol should be accompanied by tighter regulation of glycerol membrane permeability by AQGPs. Yet, since glycerol concentration is tissue-dependent, testicular glycerol concentration in individuals with or without metabolic diseases and hypogonadism is still a mystery. However, some reports show some evidence of a possible dysregulation of AQGPs-mediated glycerol permeability in the male reproductive tract of animals with metabolic syndrome [109,110]. AQGP expression is also gender-specific in adipose and hepatic tissue, indicating that AQGP expression can be dependent on sexual hormone secretion [111,112]. Hence, the potential dysregulation of AQGP-mediated glycerol permeability in men with hypogonadism can be a factor that worsens the fertility outcomes of these men.

## 6. Osmoregulation, Male Fertility, and the Effect of Hypogonadism and Metabolic Diseases

Given that osmoregulation plays a crucial role in several events that occur in the male reproductive tract (Table 1), such as spermatogenesis [113] and sperm motility [92,114], we hypothesized if hormonal regulation of AQPs participates in the regulatory mechanisms that link the metabolic status and sexual/reproductive capacity. Glycerol functions can be seen as possible evidence supporting this hypothesis since when in physiological concentrations this organic compound is considered a key factor in maintaining the spermatogenic process; however, when present in high levels, it becomes toxic to the point of being able to disrupt the blood-testis barrier thus, causing oligospermia or even azoospermia [106]. Furthermore, insulin and leptin have been shown to mediate AQGPs activity in human hepatocytes and adipocytes via the PI3K/Akt/mTOR signaling cascade [90], which is involved in many male reproductive processes such as HPG control, spermatogonia development as well as the defense from possible pathogens [115].

The literature highlights that different AQPs have specific expression patterns in the male reproductive tract. For instance, AQP3 is expressed throughout the human body [93], being found in male sex organs such as the prostate, seminal vesicles, and epididymis, and in other cells of the reproductive tract such as spermatozoa and SCs [116]. AQP7 is also found in different cell types of the male reproductive tract, although it has a different expression pattern from the one of AQP3 [117,118]. The absence of AQP7 may induce abnormalities in terms of sperm motility and morphology, ultimately leading to infertility [93]. Another relevant example to mention in this context is AQP9, which has stood out in the last few years. Similar to AQP3 and AQP7, this protein is also permeable to glycerol [119]. However, perhaps because of the wider width of the channel hole, it allows the passage of other solutes such as acetate and lactate [120]. Meanwhile, the expression of this membrane transporter was described in spermatocytes, germinal epithelium, SCs, epididymis, efferent ducts, and vas deferens [94,121]. On the other hand, the same is not true for human spermatozoa, which apparently do not exhibit traces of AQP9 [121]. Nevertheless, the expression pattern exhibited by AQGPs suggests that it is considered important for male fertility, with putative roles on fluid regulation in the male reproductive tract [93] and sperm volume regulation [92,122].

Some reports suggest that androgens have a role in the expression patterns of some AQPs thus, possibly interfering with the fluid homeodynamic of the male reproductive tract [97,98,99,100]. One of the first reports on this was by Pastor-Soler et al.; their work showed that the castration of rats decreased the levels of AQP9 expression in the epithelial cells of the epididymis in relation to control rats. Interestingly, treatment of castrated rats with physiological levels of T restored the AQP9 expression levels and pattern to those seen in control rats [97]. Similar results to the ones described in the previous study were also found in rat ventral prostate tissue [98]. Another work took a different approach to the matter and arrived at a different conclusion. This work stated that the AQP9 expression pattern in rat epithelial epididymis is androgen-independent because the expression was not modulated by postnatal development and its consequent increase in androgen levels. This result can indicate that T may not be the only hormone that regulates AQP9 expression in the rat epididymis [99]. Despite that, the relation between androgens and AQP9 was further strengthened in an experiment with castrated rats and androgen treatment (DHT). Once again, rat castration decreased AQP9 expression in the epithelial cells of the epididymis and rete testis but not from AQP1. Androgen treatment restored AQP9 levels but presented no effect on AQP1 expression levels [100]. This study points to the fact that not all AQPs are sex hormone-dependent as seen for the AQP9.

The epithelial cells of the epididymis are responsible for the reabsorption of most of the fluid that helps the spermatozoa transport from the rete testis into the efferent ducts and epididymis [123]. It is also known that AQP9 has an important role in glycerol concentration in the epididymal lumen, which is believed to have a role in sperm maturation [95,105]. Furthermore, most mammals have the osmolarity of the epididymal fluid increasing from the first epididymal section to the last [124]. That could indicate that different osmolarities are needed for different stages of sperm maturation. Thus, fluid homeodynamics in the epithelial cells of the epididymis (mostly regulated by AQPs) is vital for sperm maturation. If AQP9 expression is androgen dependent, the dysregulation of AQP9 permeability in men suffering from hypogonadism can be a factor that worsens their fertility outcomes. Yet another work that studied the expression pattern of the different AQPs during the spermatogenic phases of teleost, showed that AQP expression during spermatogenesis has both androgen-dependent and androgen-independent pathways [125]. It is, however, worth mentioning that teleost AQPs are homologs to the ones found in mammals, presenting different structures. Thus, its regulation mechanisms can also not be translatable to humans. Despite that, most of the literature on the subject seems to be in concordance with the involvement of androgens in AQP9 expression.

On the other end of the spectrum, estrogens also seem to modulate AQP expression. Interestingly, as in the works where the effect of androgens was studied, AQP9 seems to be the most responsive to sex hormones and the one that demands more interest from investigators. In cultured mice SCs, Aqp9 mRNA expression was found to decrease after estrogen treatment, accompanied by a decrease in glycerol permeability of those cells [35]. If that would be directly translated to decrease AQP9-mediated diffusion in in vivo situations, it could present a problem for the proper development of spermatogenesis. AQP9 is also permeable to lactate, which is the main energy substrate for developing germ cells [96,106]. Impairment in the supply of this metabolite from the SCs to the developing germ cells hampers the spermatogenic process. However, when a similar treatment is performed in in vivo studies, the effects seem to be the opposite. Treatment of neonatal rats with an estrogen-like drug (diethylstilbestrol) resulted in tripled AQP9 expression in the efferent ducts [126]. Nonetheless, this study also stated that this response is tissue-specific, meaning that the same stimulus has a different impact on AQP9 expression in different tissues [99]. It is also worth mentioning that a stimulus with supra-physiological estrogen levels is more effective in developing males than in adult subjects [101]. In castrated adult rats, an androgen metabolite with estrogenic effects (3-β-diol) showed to restore AQP9 expression after the decrease caused by castration [102]. This highlights that both androgens and estrogens are needed for physiological AQP9 expression patterns [100,102]. It is known that estrogens can be synthesized from androgens. This is particularly common in men with metabolic syndrome by T aromatization in adipose tissue [127]. That creates a vicious cycle and possible development of late-onset hypogonadism with a consequent negative impact on male fertility [20].

The studies presented indicate that sub-physiological levels of androgens negatively impact AQP9 expression and that increasing sex hormones to healthy levels restores AQP9 expression levels in cells of the male reproductive tract in in vivo rats. Nevertheless, it remains to be clarified the effect on AQP9 expression levels of individuals with metabolic syndrome (and consequent dysregulation of androgen and estrogens) in comparison to normal individuals with physiological levels of sex hormones. Rats with metabolic syndrome induced by high-fat consumption presented higher Aqp9 expression in the testis and epididymis, despite presenting lower T but higher estrogen levels when compared to the control group [109]. Thus, increased AQP9 expression and supra-physiological AQP9-mediated permeability of solutes like glycerol can be deleterious for sperm formation and male fertility. This theory gains an interesting twist by the fact that an opposite expression pattern is seen for AQP1 (an orthodox AQP) [109]. The overexpression of AQP9, which permeates glycerol and other solutes, contrasts with the lower expression of AQP1, which is known to be one of the most permeable to water. However, this topic is still scarcely investigated, and more studies should be made to clarify the importance of testosterone on AQP-mediated reproductive tract permeability and male fertility.

## 7. Conclusions and Future Perspectives

It is critical to underline the relevance of the male gender in infertility, as well as the function of T in this shift of male sexual healthiness into sickness. During the last few years, enormous progress has been achieved in the understanding and treatment of T-related hormonal abnormalities in males. However, as discussed, fully understanding this scenario is still far. Notwithstanding, the involvement of AQPs in the development of male fertility in hypogonadal men is becoming apparent. Many unanswered questions remain for the scientific community to investigate, including the impact of medications, the usage of long-term therapy, and the pathological network underpinning T imbalance. Furthermore, as metabolic diseases among young people become more prevalent, the potential for male reproductive health deterioration grows. As a result, there is a growing need to invest in potential screening methods and drug administration, maybe by looking into the impacts of AQPs in this pathological scenario. Additionally, it may be beneficial to have a better understanding of the viability and potential economic benefits of pharmaceutical assembly. As a result, it is self-evident that gathering new data in this direction would improve the efficiency with which infertility is dealt with, addressing the scientific gaps that exist in present times.

## Figures and Tables

**Table 1:** Aquaporin (AQP)-3, 7, and 9 functions on metabolism and in male fertility.

Aquaporins	Metabolic Function	Fertility Function	References
AQP3	Adipocyte glycerol efflux		[10]
	Upregulated by insulin	Sperm osmoadaptation	[90,92]
	Upregulated by leptin		[90]
AQP7	Adipocyte glycerol efflux		[88,89]
	Upregulated by insulin	Sperm motility	[90,93]
	Downregulated by leptin	Spermiogenesis	[90,93]
	Increases insulin sensitivity		[91]
AQP9	Adipocyte glycerol influx	Spermatogenesis	[10,94]
	Hepatocyte glycerol influx	Regulation of epididymal osmolarity	[11,93,95]
	Upregulated by insulin	Lactate secretion for germ cells	[90,96]
	Downregulated by leptin	Upregulated by sex hormones	[35,90,97,98,99,100,101,102]

## Data Availability

No new data were created or analyzed in this study. Data sharing is not applicable to this article.

## References

[1] Zegers-Hochschild F., Adamson G.D., de Mouzon J., Ishihara O., Mansour R., Nygren K., Sullivan E., Vanderpoel S. (2009). International Committee for Monitoring Assisted Reproductive Technology (ICMART) and the World Health Organization (WHO) revised glossary of ART terminology, 2009. Fertil. Steril..

[2] Health T.L.G. (2022). Infertility—Why the silence?. Lancet Glob. Health.

[3] Agarwal A., Mulgund A., Hamada A., Chyatte M.R. (2015). A unique view on male infertility around the globe. Reprod. Biol. Endocrinol..

[4] Qi X., Wang K., Zhou G., Xu Z., Yu J., Zhang W. (2016). The role of testicular artery in laparoscopic varicocelectomy: A systematic review and meta-analysis. Int. Urol. Nephrol..

[5] Finkelstein J.S., Lee H., Burnett-Bowie S.A., Pallais J.C., Yu E.W., Borges L.F., Jones B.F., Barry C.V., Wulczyn K.E., Thomas B.J. (2013). Gonadal steroids and body composition, strength, and sexual function in men. N. Engl. J. Med..

[6] Crisóstomo L., Pereira S.C., Monteiro M.P., Raposo J.F., Oliveira P.F., Alves M.G. (2020). Lifestyle, metabolic disorders and male hypogonadism—A one-way ticket?. Mol. Cell. Endocrinol..

[7] Khera M., Broderick G.A., Carson C.C., Dobs A.S., Faraday M.M., Goldstein I., Hakim L.S., Hellstrom W.J., Kacker R., Köhler T.S. (2016). Adult-Onset Hypogonadism. Mayo Clin. Proc..

[8] Morales A., Lunenfeld B. (2002). Investigation, treatment and monitoring of late-onset hypogonadism in males. Official Recommendations of ISSAM. Aging Male.

[9] Wang C., Nieschlag E., Swerdloff R., Behre H.M., Hellstrom W.J., Gooren L.J., Kaufman J.M., Legros J.J., Lunenfeld B., Morales A. (2009). ISA, ISSAM, EAU, EAA and ASA recommendations: Investigation, treatment and monitoring of late-onset hypogonadism in males. Int. J. Impot. Res..

[10] Rodríguez A., Catalán V., Gómez-Ambrosi J., Frühbeck G. (2011). Aquaglyceroporins serve as metabolic gateways in adiposity and insulin resistance control. Cell Cycle.

[11] Rodríguez A., Gena P., Méndez-Giménez L., Rosito A., Valentí V., Rotellar F., Sola I., Moncada R., Silva C., Svelto M. (2014). Reduced hepatic aquaporin-9 and glycerol permeability are related to insulin resistance in non-alcoholic fatty liver disease. Int. J. Obes..

[12] Verkman A.S. (2012). Aquaporins in clinical medicine. Annu. Rev. Med..

[13] Ribeiro J.C., Alves M.G., Yeste M., Cho Y.S., Calamita G., Oliveira P.F. (2021). Aquaporins and (in)fertility: More than just water transport. Biochim. Biophys. Acta Mol. Basis Dis..

[14] Bienenfeld A., Azarchi S., Lo Sicco K., Marchbein S., Shapiro J., Nagler A.R. (2019). Androgens in women: Androgen-mediated skin disease and patient evaluation. J. Am. Acad. Dermatol..

[15] Rato L., Alves M.G., Socorro S., Duarte A.I., Cavaco J.E., Oliveira P.F. (2012). Metabolic regulation is important for spermatogenesis. Nat. Rev. Urol..

[16] Alves M.G., Rato L., Carvalho R.A., Moreira P.I., Socorro S., Oliveira P.F. (2013). Hormonal control of Sertoli cell metabolism regulates spermatogenesis. Cell. Mol. Life Sci..

[17] Walker W.H., Cheng J. (2005). FSH and testosterone signaling in Sertoli cells. Reproduction.

[18] Schulster M., Bernie A.M., Ramasamy R. (2016). The role of estradiol in male reproductive function. Asian J. Androl..

[19] Nestler E.J., Hyman S.E., Holtzman D.M., Malenka R.C. (2015). Neural and Neuroendocrine Control of the Internal Milieu. Molecular Neuropharmacology: A Foundation for Clinical Neuroscience, 3 ed..

[20] Braga P.C., Pereira S.C., Ribeiro J.C., Sousa M., Monteiro M.P., Oliveira P.F., Alves M.G. (2020). Late-onset hypogonadism and lifestyle-related metabolic disorders. Andrology.

[21] Blair J.A., McGee H., Bhatta S., Palm R., Casadesus G. (2015). Hypothalamic-pituitary-gonadal axis involvement in learning and memory and Alzheimer’s disease: More than “just” estrogen. Front. Endocrinol..

[22] Pinilla L., Aguilar E., Dieguez C., Millar R.P., Tena-Sempere M. (2012). Kisspeptins and reproduction: Physiological roles and regulatory mechanisms. Physiol. Rev..

[23] Roa J., Aguilar E., Dieguez C., Pinilla L., Tena-Sempere M. (2008). New frontiers in kisspeptin/GPR54 physiology as fundamental gatekeepers of reproductive function. Front. Neuroendocrinol..

[24] Basaria S. (2014). Male hypogonadism. Lancet.

[25] Weinbauer G., Luetjens C.M., Simoni M., Nieschlag E., Nieschlag E., Behre H.M., Nieschlag S. (2010). Physiology of Testicular Function. Andrology: Male Reproductive Health and Dysfunction.

[26] Tilbrook A.J., Clarke I.J. (2001). Negative feedback regulation of the secretion and actions of gonadotropin-releasing hormone in males. Biol. Reprod..

[27] Sharpe R. (1994). Regulation of spermatogenesis. Physiol. Reprod..

[28] Niederberger C. (2011). An Introduction to Male Reproductive Medicine.

[29] Lamm S., Chidakel A., Bansal R. (2016). Obesity and Hypogonadism. Urol. Clin. N. Am..

[30] Skorupskaite K., George J.T., Anderson R.A. (2014). The kisspeptin-GnRH pathway in human reproductive health and disease. Hum. Reprod. Update.

[31] Walker W.H. (2010). Non-classical actions of testosterone and spermatogenesis. Philos. Trans. R. Soc. B Biol. Sci.

[32] Walker W.H. (2009). Molecular mechanisms of testosterone action in spermatogenesis. Steroids.

[33] Walker W.H. (2011). Testosterone signaling and the regulation of spermatogenesis. Spermatogenesis.

[34] Fix C., Jordan C., Cano P., Walker W.H. (2004). Testosterone activates mitogen-activated protein kinase and the cAMP response element binding protein transcription factor in Sertoli cells. Proc. Natl. Acad. Sci. USA.

[35] Bernardino R.L., Carrageta D.F., Silva A.M., Calamita G., Alves M.G., Soveral G., Oliveira P.F. (2018). Estrogen Modulates Glycerol Permeability in Sertoli Cells through Downregulation of Aquaporin-9. Cells.

[36] Ishikawa T., Fujioka H., Ishimura T., Takenaka A., Fujisawa M. (2007). Expression of leptin and leptin receptor in the testis of fertile and infertile patients. Andrologia.

[37] Giovambattista A., Suescun M.O., Nessralla C.C., França L.R., Spinedi E., Calandra R.S. (2003). Modulatory effects of leptin on leydig cell function of normal and hyperleptinemic rats. Neuroendocrinology.

[38] Quigley C.A., De Bellis A., Marschke K.B., el-Awady M.K., Wilson E.M., French F.S. (1995). Androgen receptor defects: Historical, clinical, and molecular perspectives. Endocr. Rev..

[39] Dohle G., Arver S., Bettocchi C., Kliesch S., Punab M., De Ronde W., Urology E.A. (2012). Guidelines on male hypogonadism. Eur. Assoc. Urol..

[40] Ahern T., Swiecicka A., Eendebak R.J.A.H., Carter E.L., Finn J.D., Pye S.R., O’Neill T.W., Antonio L., Keevil B., Bartfai G. (2016). Natural history, risk factors and clinical features of primary hypogonadism in ageing men: Longitudinal Data from the European Male Ageing Study. Clin. Endocrinol..

[41] Ventimiglia E., Ippolito S., Capogrosso P., Pederzoli F., Cazzaniga W., Boeri L., Cavarretta I., Alfano M., Viganò P., Montorsi F. (2017). Primary, secondary and compensated hypogonadism: A novel risk stratification for infertile men. Andrology.

[42] Fraietta R., Zylberstejn D.S., Esteves S.C. (2013). Hypogonadotropic hypogonadism revisited. Clinics.

[43] Tajar A., Forti G., O’Neill T.W., Lee D.M., Silman A.J., Finn J.D., Bartfai G., Boonen S., Casanueva F.F., Giwercman A. (2010). Characteristics of secondary, primary, and compensated hypogonadism in aging men: Evidence from the European Male Ageing Study. J. Clin. Endocrinol. Metab..

[44] Veldhuis J.D., Liu P.Y., Keenan D.M., Takahashi P.Y. (2012). Older men exhibit reduced efficacy of and heightened potency downregulation by intravenous pulses of recombinant human LH: A study in 92 healthy men. Am. J. Physiol. Endocrinol. Metab..

[45] Gooren L. (2009). Late-onset hypogonadism. Front. Horm. Res..

[46] Bhasin S., Brito J.P., Cunningham G.R., Hayes F.J., Hodis H.N., Matsumoto A.M., Snyder P.J., Swerdloff R.S., Wu F.C., Yialamas M.A. (2018). Testosterone Therapy in Men With Hypogonadism: An Endocrine Society Clinical Practice Guideline. J. Clin. Endocrinol. Metab..

[47] Belloc S., Cohen-Bacrie M., Amar E., Izard V., Benkhalifa M., Dalléac A., de Mouzon J. (2014). High body mass index has a deleterious effect on semen parameters except morphology: Results from a large cohort study. Fertil. Steril..

[48] Hammiche F., Laven J.S., Twigt J.M., Boellaard W.P., Steegers E.A., Steegers-Theunissen R.P. (2012). Body mass index and central adiposity are associated with sperm quality in men of subfertile couples. Hum. Reprod..

[49] Luque E.M., Tissera A., Gaggino M.P., Molina R.I., Mangeaud A., Vincenti L.M., Beltramone F., Larcher J.S., Estofán D., Fiol de Cuneo M. (2017). Body mass index and human sperm quality: Neither one extreme nor the other. Reprod. Fertil. Dev..

[50] Alves M.G., Jesus T.T., Sousa M., Goldberg E., Silva B.M., Oliveira P.F. (2016). Male fertility and obesity: Are ghrelin, leptin and glucagon-like peptide-1 pharmacologically relevant?. Curr. Pharm. Des..

[51] Crisóstomo L., Alves M.G., Gorga A., Sousa M., Riera M.F., Galardo M.N., Meroni S.B., Oliveira P.F. (2018). Molecular Mechanisms and Signaling Pathways Involved in the Nutritional Support of Spermatogenesis by Sertoli Cells. Sertoli Cells.

[52] Jesus T.T., Oliveira P.F., Sousa M., Cheng C.Y., Alves M.G. (2017). Mammalian target of rapamycin (mTOR): A central regulator of male fertility?. Crit. Rev. Biochem. Mol. Biol..

[53] Monteiro M.P., Batterham R.L. (2017). The Importance of the Gastrointestinal Tract in Controlling Food Intake and Regulating Energy Balance. Gastroenterology.

[54] Rato L., Meneses M.J., Silva B.M., Sousa M., Alves M.G., Oliveira P.F. (2016). New insights on hormones and factors that modulate Sertoli cell metabolism. Histol. Histopathol..

[55] Carrageta D.F., Oliveira P.F., Alves M.G., Monteiro M.P. (2019). Obesity and male hypogonadism: Tales of a vicious cycle. Obes. Rev..

[56] Singh A.B., Hsia S., Alaupovic P., Sinha-Hikim I., Woodhouse L., Buchanan T.A., Shen R., Bross R., Berman N., Bhasin S. (2002). The effects of varying doses of T on insulin sensitivity, plasma lipids, apolipoproteins, and C-reactive protein in healthy young men. J. Clin. Endocrinol. Metab..

[57] Grossmann M. (2014). Testosterone and glucose metabolism in men: Current concepts and controversies. J. Endocrinol..

[58] Singh R., Artaza J.N., Taylor W.E., Gonzalez-Cadavid N.F., Bhasin S. (2003). Androgens stimulate myogenic differentiation and inhibit adipogenesis in C3H 10T1/2 pluripotent cells through an androgen receptor-mediated pathway. Endocrinology.

[59] McInnes K.J., Smith L.B., Hunger N.I., Saunders P.T., Andrew R., Walker B.R. (2012). Deletion of the androgen receptor in adipose tissue in male mice elevates retinol binding protein 4 and reveals independent effects on visceral fat mass and on glucose homeostasis. Diabetes.

[60] Yanase T., Fan W., Kyoya K., Min L., Takayanagi R., Kato S., Nawata H. (2008). Androgens and metabolic syndrome: Lessons from androgen receptor knock out (ARKO) mice. J. Steroid Biochem. Mol. Biol..

[61] Kelly D.M., Jones T.H. (2015). Testosterone and obesity. Obes. Rev..

[62] Rebuffé-Scrive M., Mårin P., Björntorp P. (1991). Effect of testosterone on abdominal adipose tissue in men. Int. J. Obes..

[63] Olivecrona G. (2016). Role of lipoprotein lipase in lipid metabolism. Curr. Opin. Lipidol..

[64] Ramirez M.E., McMurry M.P., Wiebke G.A., Felten K.J., Ren K., Meikle A.W., Iverius P.H. (1997). Evidence for sex steroid inhibition of lipoprotein lipase in men: Comparison of abdominal and femoral adipose tissue. Metabolism.

[65] Mårin P., Lönn L., Andersson B., Odén B., Olbe L., Bengtsson B.A., Björntorp P. (1996). Assimilation of triglycerides in subcutaneous and intraabdominal adipose tissues in vivo in men: Effects of testosterone. J. Clin. Endocrinol. Metab..

[66] Corona G., Mannucci E., Ricca V., Lotti F., Boddi V., Bandini E., Balercia G., Forti G., Maggi M. (2009). The age-related decline of testosterone is associated with different specific symptoms and signs in patients with sexual dysfunction. Int. J. Androl..

[67] Allen N.E., Appleby P.N., Davey G.K., Key T.J. (2002). Lifestyle and nutritional determinants of bioavailable androgens and related hormones in British men. Cancer Causes Control.

[68] Gapstur S.M., Gann P.H., Kopp P., Colangelo L., Longcope C., Liu K. (2002). Serum androgen concentrations in young men: A longitudinal analysis of associations with age, obesity, and race. The CARDIA male hormone study. Cancer Epidemiol. Biomark. Prev..

[69] Jensen T.K., Andersson A.M., Jørgensen N., Andersen A.G., Carlsen E., Petersen J.H., Skakkebaek N.E. (2004). Body mass index in relation to semen quality and reproductive hormones among 1,558 Danish men. Fertil. Steril..

[70] Svartberg J., von Mühlen D., Sundsfjord J., Jorde R. (2004). Waist circumference and testosterone levels in community dwelling men. The Tromsø study. Eur. J. Epidemiol..

[71] Svartberg J., von Mühlen D., Schirmer H., Barrett-Connor E., Sundfjord J., Jorde R. (2004). Association of endogenous testosterone with blood pressure and left ventricular mass in men. The Tromsø Study. Eur. J. Endocrinol..

[72] Corona G., Rastrelli G., Monami M., Saad F., Luconi M., Lucchese M., Facchiano E., Sforza A., Forti G., Mannucci E. (2013). Body weight loss reverts obesity-associated hypogonadotropic hypogonadism: A systematic review and meta-analysis. Eur. J. Endocrinol..

[73] Farias J.M., Tinetti M., Khoury M., Umpierrez G.E. (2014). Low testosterone concentration and atherosclerotic disease markers in male patients with type 2 diabetes. J. Clin. Endocrinol. Metab..

[74] Mohammadi M., Gozashti M.H., Aghadavood M., Mehdizadeh M.R., Hayatbakhsh M.M. (2017). Clinical Significance of Serum IL-6 and TNF-α Levels in Patients with Metabolic Syndrome. Rep. Biochem. Mol. Biol..

[75] Kern P.A., Ranganathan S., Li C., Wood L., Ranganathan G. (2001). Adipose tissue tumor necrosis factor and interleukin-6 expression in human obesity and insulin resistance. Am. J. Physiol.-Endocrinol. Metab..

[76] Lainampetch J., Panprathip P., Phosat C., Chumpathat N., Prangthip P., Soonthornworasiri N., Puduang S., Wechjakwen N., Kwanbunjan K. (2019). Association of Tumor Necrosis Factor Alpha, Interleukin 6, and C-Reactive Protein with the Risk of Developing Type 2 Diabetes: A Retrospective Cohort Study of Rural Thais. J. Diabetes Res..

[77] Roseweir A.K., Millar R.P. (2009). The role of kisspeptin in the control of gonadotrophin secretion. Hum. Reprod. Update.

[78] Isidori A.M., Caprio M., Strollo F., Moretti C., Frajese G., Isidori A., Fabbri A. (1999). Leptin and androgens in male obesity: Evidence for leptin contribution to reduced androgen levels. J. Clin. Endocrinol. Metab..

[79] Page S.T., Herbst K.L., Amory J.K., Coviello A.D., Anawalt B.D., Matsumoto A.M., Bremner W.J. (2005). Testosterone administration suppresses adiponectin levels in men. J. Androl..

[80] Kapoor D., Clarke S., Stanworth R., Channer K.S., Jones T.H. (2007). The effect of testosterone replacement therapy on adipocytokines and C-reactive protein in hypogonadal men with type 2 diabetes. Eur. J. Endocrinol..

[81] Lanfranco F., Zitzmann M., Simoni M., Nieschlag E. (2004). Serum adiponectin levels in hypogonadal males: Influence of testosterone replacement therapy. Clin. Endocrinol..

[82] Elsaied M.A., Masallat D., Abdel-Hamid I.A. (2019). Correlation of Adiponectin With Testosterone in Patients With and Without Type 2 Diabetes and Erectile Dysfunction. Am. J. Men’s Health.

[83] Ishibashi K., Hara S., Kondo S. (2009). Aquaporin water channels in mammals. Clin. Exp. Nephrol..

[84] Verkman A.S. (2005). More than just water channels: Unexpected cellular roles of aquaporins. J. Cell Sci..

[85] Rojek A., Praetorius J., Frøkiaer J., Nielsen S., Fenton R.A. (2008). A Current View of the Mammalian Aquaglyceroporins. Annu. Rev. Physiol..

[86] Agre P. (2004). Aquaporin Water Channels (Nobel Lecture). Angew. Chem. Int. Ed..

[87] Calamita G., Perret J., Delporte C. (2018). Aquaglyceroporins: Drug Targets for Metabolic Diseases?. Front. Physiol..

[88] Hibuse T., Maeda N., Funahashi T., Yamamoto K., Nagasawa A., Mizunoya W., Kishida K., Inoue K., Kuriyama H., Nakamura T. (2005). Aquaporin 7 deficiency is associated with development of obesity through activation of adipose glycerol kinase. Proc. Natl. Acad. Sci. USA.

[89] MacDougald O.A., Burant C.F. (2005). Obesity and metabolic perturbations after loss of aquaporin 7, the adipose glycerol transporter. Proc. Natl. Acad. Sci. USA.

[90] Rodríguez A., Catalán V., Gómez-Ambrosi J., García-Navarro S., Rotellar F., Valentí V., Silva C., Gil M.J., Salvador J., Burrell M.A. (2011). Insulin- and leptin-mediated control of aquaglyceroporins in human adipocytes and hepatocytes is mediated via the PI3K/Akt/mTOR signaling cascade. J. Clin. Endocrinol. Metab..

[91] Shen F.X., Gu X., Pan W., Li W.P., Li W., Ye J., Yang L.J., Gu X.J., Ni L.S. (2012). Over-expression of AQP7 contributes to improve insulin resistance in adipocytes. Exp. Cell Res..

[92] Chen Q., Peng H., Lei L., Zhang Y., Kuang H., Cao Y., Shi Q.X., Ma T., Duan E. (2011). Aquaporin3 is a sperm water channel essential for postcopulatory sperm osmoadaptation and migration. Cell Res..

[93] Carrageta D.F., Bernardino R.L., Soveral G., Calamita G., Alves M.G., Oliveira P.F. (2020). Aquaporins and male (in)fertility: Expression and role throughout the male reproductive tract. Arch. Biochem. Biophys..

[94] Pastor-Soler N., Bagnis C., Sabolic I., Tyszkowski R., McKee M., Van Hoek A., Breton S., Brown D. (2001). Aquaporin 9 expression along the male reproductive tract. Biol. Reprod..

[95] Arrighi S. (2014). Are the basal cells of the mammalian epididymis still an enigma?. Reprod. Fertil. Dev..

[96] Arena S., Arena F., Maisano D., Di Benedetto V., Romeo C., Nicòtina P.A. (2011). Aquaporin-9 immunohistochemistry in varicocele testes as a consequence of hypoxia in the sperm production site. Andrologia.

[97] Pastor-Soler N., Isnard-Bagnis C., Herak-Kramberger C., Sabolic I., Van Hoek A., Brown D., Breton S. (2002). Expression of Aquaporin 9 in the Adult Rat Epididymal Epithelium Is Modulated by Androgens. Biol. Reprod..

[98] Wang J., Tanji N., Sasaki T., Kikugawa T., Song X., Yokoyama M. (2008). Androgens upregulate aquaporin 9 expression in the prostate. Int. J. Urol..

[99] Badran H.H., Hermo L.S. (2002). Expression and regulation of aquaporins 1, 8, and 9 in the testis, efferent ducts, and epididymis of adult rats and during postnatal development. J. Androl..

[100] Oliveira C.A., Carnes K., França L.R., Hermo L., Hess R.A. (2005). Aquaporin-1 and -9 are differentially regulated by oestrogen in the efferent ductule epithelium and initial segment of the epididymis. Biol. Cell.

[101] Aksglaede L., Juul A., Leffers H., Skakkebaek N.E., Andersson A.M. (2006). The sensitivity of the child to sex steroids: Possible impact of exogenous estrogens. Hum. Reprod. Update.

[102] Picciarelli-Lima P., Oliveira A.G., Reis A.M., Kalapothakis E., Mahecha G.A.B., Hess R.A., Oliveira C.A. (2006). Effects of 3-beta-diol, an androgen metabolite with intrinsic estrogen-like effects, in modulating the aquaporin-9 expression in the rat efferent ductules. Reprod. Biol. Endocrinol..

[103] Liu Y., Promeneur D., Rojek A., Kumar N., Frøkiær J., Nielsen S., King L.S., Agre P., Carbrey J.M. (2007). Aquaporin 9 is the major pathway for glycerol uptake by mouse erythrocytes, with implications for malarial virulence. Proc. Natl. Acad. Sci. USA.

[104] Pimpão C., Wragg D., da Silva I.V., Casini A., Soveral G. (2022). Aquaglyceroporin Modulators as Emergent Pharmacological Molecules for Human Diseases. Front. Mol. Biosci..

[105] Cooper T.G., Brooks D.E. (1981). Entry of glycerol into the rat epididymis and its utilization by epididymal spermatozoa. Reproduction.

[106] Crisóstomo L., Alves M.G., Calamita G., Sousa M., Oliveira P.F. (2017). Glycerol and testicular activity: The good, the bad and the ugly. Mol. Hum. Reprod..

[107] Arner P., Rydén M. (2015). Fatty Acids, Obesity and Insulin Resistance. Obes. Facts.

[108] Skowronski R., Hollenbeck C.B., Varasteh B.B., Chen Y.D., Reaven G.M. (1991). Regulation of non-esterified fatty acid and glycerol concentration by insulin in normal individuals and patients with type 2 diabetes. Diabet. Med..

[109] Marchiani S., Vignozzi L., Filippi S., Gurrieri B., Comeglio P., Morelli A., Danza G., Bartolucci G., Maggi M., Baldi E. (2015). Metabolic syndrome-associated sperm alterations in an experimental rabbit model: Relation with metabolic profile, testis and epididymis gene expression and effect of tamoxifen treatment. Mol. Cell. Endocrinol..

[110] Pei L., Yang G., Jiang J., Jiang R., Deng Q., Chen B., Gan X. (2013). Expression of aquaporins in prostate and seminal vesicles of diabetic rats. J. Sex. Med..

[111] Lebeck J., Østergård T., Rojek A., Füchtbauer E.M., Lund S., Nielsen S., Praetorius J. (2012). Gender-specific effect of physical training on AQP7 protein expression in human adipose tissue. Acta Diabetol..

[112] Rodríguez A., Marinelli R.A., Tesse A., Frühbeck G., Calamita G. (2015). Sexual Dimorphism of Adipose and Hepatic Aquaglyceroporins in Health and Metabolic Disorders. Front. Endocrinol..

[113] Yang B., Song Y., Zhao D., Verkman A.S. (2005). Phenotype analysis of aquaporin-8 null mice. Am. J. Physiol. Cell Physiol..

[114] Saito K., Kageyama Y., Okada Y., Kawakami S., Kihara K., Ishibashi K., Sasaki S. (2004). Localization of aquaporin-7 in human testis and ejaculated sperm: Possible involvement in maintenance of sperm quality. J. Urol..

[115] Deng C.Y., Lv M., Luo B.H., Zhao S.Z., Mo Z.C., Xie Y.J. (2021). The Role of the PI3K/AKT/mTOR Signalling Pathway in Male Reproduction. Curr. Mol. Med..

[116] Mobasheri A., Wray S., Marples D. (2005). Distribution of AQP2 and AQP3 water channels in human tissue microarrays. J. Mol. Histol..

[117] Laforenza U., Pellavio G., Marchetti A.L., Omes C., Todaro F., Gastaldi G. (2016). Aquaporin-Mediated Water and Hydrogen Peroxide Transport Is Involved in Normal Human Spermatozoa Functioning. Int. J. Mol. Sci..

[118] Moretti E., Terzuoli G., Mazzi L., Iacoponi F., Collodel G. (2012). Immunolocalization of aquaporin 7 in human sperm and its relationship with semen parameters. Syst. Biol. Reprod. Med..

[119] Yeste M., Morató R., Rodríguez-Gil J.E., Bonet S., Prieto-Martínez N. (2017). Aquaporins in the male reproductive tract and sperm: Functional implications and cryobiology. Reprod. Domest. Anim..

[120] Badaut J., Regli L. (2004). Distribution and possible roles of aquaporin 9 in the brain. Neuroscience.

[121] Yeung C.H., Callies C., Tüttelmann F., Kliesch S., Cooper T.G. (2010). Aquaporins in the human testis and spermatozoa—Identification, involvement in sperm volume regulation and clinical relevance. Int. J. Androl..

[122] Chen Q., Duan E.K. (2011). Aquaporins in sperm osmoadaptation: An emerging role for volume regulation. Acta Pharmacol. Sin..

[123] James E.R., Carrell D.T., Aston K.I., Jenkins T.G., Yeste M., Salas-Huetos A. (2020). The Role of the Epididymis and the Contribution of Epididymosomes to Mammalian Reproduction. Int. J. Mol. Sci..

[124] Yeung C.H., Barfield J.P., Cooper T.G. (2006). Physiological volume regulation by spermatozoa. Mol. Cell. Endocrinol..

[125] Boj M., Chauvigné F., Zapater C., Cerdà J. (2015). Gonadotropin-Activated Androgen-Dependent and Independent Pathways Regulate Aquaporin Expression during Teleost (Sparus aurata) Spermatogenesis. PLoS ONE.

[126] Wellejus A., Jensen H.E., Loft S., Jonassen T.E. (2008). Expression of aquaporin 9 in rat liver and efferent ducts of the male reproductive system after neonatal diethylstilbestrol exposure. J. Histochem. Cytochem..

[127] Polari L., Yatkin E., Martínez Chacón M.G., Ahotupa M., Smeds A., Strauss L., Zhang F., Poutanen M., Saarinen N., Mäkelä S.I. (2015). Weight gain and inflammation regulate aromatase expression in male adipose tissue, as evidenced by reporter gene activity. Mol. Cell. Endocrinol..

